# The Second Hit Hypothesis in Animal and Human Dystonia: The Role of Peripheral Nerve Trauma and Spinal Cord Injury

**DOI:** 10.1002/mds.70087

**Published:** 2025-10-14

**Authors:** Lisa Harder‐Rauschenberger, Chi Wang Ip

**Affiliations:** ^1^ Department of Neurology University Hospital of Würzburg Würzburg Germany

**Keywords:** dystonia, gene‐environment interaction, nerve injury, second hit, spinal cord injury

## Abstract

Dystonia is a complex movement disorder characterized by involuntary muscle contractions and abnormal postures. Although genetic factors have been implicated in dystonia pathogenesis, recent evidence suggests that additional environmental triggers, referred to as the second hit, may play a significant role in the development and progression of the disease. A remarkably low penetrance in some of the monogenic forms of dystonia supports the need for additional triggers to unmask the phenotype. Given that dystonia has been reported to develop after traumatic events, this review explores the second hit hypothesis in animal models of dystonia and its potential relevance to human dystonia, with particular emphasis on the role of nerve and spinal cord injuries. These injuries trigger significant peripheral changes and profound brain and spinal cord circuit alterations, which require a healthy immune system and functional and structural plasticity responses. We discuss how nerve and spinal cord injuries initiate these key pathomechanistic processes, including neuroinflammation and the reorganization of the central sensorimotor network, which entails adaptive modifications in neural pathways to compensate for the injury. We further highlight future challenges and potential therapeutic implications of nerve and spinal cord injury–induced dystonia. Understanding the interplay between nerve injury, spinal cord injury, neuroinflammation, and dystonia may pave the way for novel therapeutic strategies targeting these factors. © 2025 The Author(s). *Movement Disorders* published by Wiley Periodicals LLC on behalf of International Parkinson and Movement Disorder Society.

## Graphical abstract

The “second‐hit” hypothesis proposes that both a genetic predisposition and an environmental insult—such as peripheral nerve trauma or spinal cord injury—are required for dystonia development. This review explores how neuroinflammation and maladaptive plasticity, triggered by nerve and spinal cord injury, contribute to dystonia pathogenesis. Created with Biorender.com.

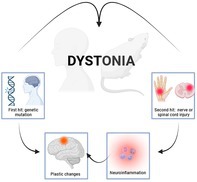

## Introduction

### Dystonia: A Complex Movement Disorder

Dystonia—the third most common movement disorder—encompasses a highly complex and heterogeneous group of disorders with strong variations in etiology and symptomatology. Dystonic symptoms are simply characterized as abnormal movements and postures resulting from involuntary muscle contractions.^1^, ^2^ However, dystonia can be early or late onset, present with varying body distributions, be accompanied by other neurological features, be genetic or acquired, and may be idiopathic or linked to nervous system pathology. Phenotypic variability in dystonia is considerable and can be noted even for the inherited forms of dystonia, such as DYT‐TOR1A dystonia.^3^, ^4^, ^5^, ^6^ Understanding dystonia pathophysiology has proven to be equally as complex as the clinical presentations of the disorder. Anatomically, multiple brain regions seem to be involved in the development of dystonia—primarily the cortex, basal ganglia, thalamus, and cerebellum.^7^, ^8^ A recent study indicated a significant role of the spinal cord in the development of DYT‐TOR1A dystonia.^9^ Widely accepted pathophysiological concepts behind dystonia include impairment of sensorimotor integration, a maladaptive plasticity, and loss of inhibition.^10^, ^11^ Striatal dopamine and acetylcholine may also play a significant role in dystonia.^12^, ^13^ Although these pathophysiological concepts are supported by solid evidence, the underlying molecular mechanisms remain unclear.

### The Second Hit Hypothesis in Dystonia

The hypothesis that extragenetic factors could contribute to the pathogenesis of dystonia emerged early. Two key observations contributed to this understanding: (1) monogenic forms of dystonia present with an incomplete penetrance; and (2) environmental factors are well‐established triggers of acute, rapid onset of dystonia in DYT/PARK‐ATP1A3 dystonia and paroxysmal dyskinesias. Concerning the first observation, reduced penetrance has been reported for most inherited forms of dystonia, eg, DYT‐TOR1A dystonia (penetrance 29%).^3^, ^14^, ^15^ Although the reduced penetrance and strong phenotypic variability in DYT‐TOR1A dystonia can partly be traced back to the presence of second genetic mutations or polymorphisms in the mutated gene, relevant studies have been heavily contradictory.^16^, ^17^, ^18^, ^19^ Concerning the second observation, physical activity, psychological stress, alcohol, caffeine, extreme temperatures, and sleep deprivation are some of the acute triggers described for DYT/PARK‐ATP1A3 and paroxysmal dyskinesias.^20^, ^21^, ^22^, ^23^ Another intimation for the role of environmental triggers comes from task‐specific dystonias—forms of dystonia described in association with the repetitive and prolonged performance of stereotyped, fine motor movements.^24^ Task‐specific dystonias have been reported in writers and typists, musicians, athletes, and many more professional fields. However, although a genetic contribution has been discussed for patients with task‐specific dystonias, no causative genes have been identified.^25^

In summary, these observations led to the proposal of a second hit or double‐hit hypothesis—ie, additional factors beyond genetic predisposition contribute to the onset and progression of dystonia development.^26^ A genetic mutation is believed to act as the first hit, leading to an endophenotype—a predisposing characteristic associated with genetic susceptibility—that heightens the vulnerability of the brain to pathological changes.^27^, ^28^, ^29^, ^30^ An extragenetic factor is hypothesized to serve as the second hit, disrupting the fragile equilibrium of the endophenotype and triggering the manifestation of dystonia. Over the years, a variety of potential extragenetic factors have been discussed and studied in the context of dystonia development. Among them are stereotyped movements, perinatal problems, infections, and cranial and extracranial injuries.^26^

### Objectives of the Review

Peripheral nerve injuries and spinal cord injuries are particularly intriguing in the context of dystonia, representing environmental factors that could disrupt neural pathways and contribute to pathological changes. Within this review, we therefore aim to explore the second hit hypothesis in dystonia, emphasizing these types of injuries as potential triggers for the condition. The neuroinflammation and remodeling of the central nervous system (CNS) underlying nerve and spinal cord injuries are discussed in relation to the pathomechanisms known to lead to dystonia. Evidence from second hit animal studies and clinical reports from patients with dystonia are analyzed. We highlight how nerve and spinal cord injury could play a significant role for dystonia development in a subgroup of patients, opening the door for new therapeutic strategies. A literature search was performed in PubMed. References were identified using the search term “dystonia” in combination with “peripheral nerve injury,” “peripheral trauma,” “posttraumatic,” “spinal cord injury,” or “neuroinflammation.” The article abstracts were carefully screened; articles not related to the topic or not written in English were excluded. Review articles relevant to our manuscript were considered. Articles were also taken from the authors’ own files.

## Pathomechanisms Behind Nerve and Spinal Cord Injuries

Peripheral nerve injuries initiate a complex cascade of events affecting both the peripheral nervous system and the CNS. In the periphery, such injuries induce Wallerian degeneration, a process characterized by degeneration of the axon distal to the injury site.^31^ Within hours to days, immune cells (such as phagocytic neutrophils, macrophages, and later on lymphocytes) infiltrate the site of injury. Peripheral axon regeneration is dependent on a healthy immune system capable of responding in a rapid and efficient manner. Indeed, depletion of macrophages led to strongly reduced axonal regeneration and inhibited recovery of motor function in mice after sciatic nerve lesion.^32^ A deficit in functional B and/or T lymphocytes led to delayed functional recovery of injured nerves.^33^, ^34^ The immune system also triggers CNS changes after peripheral nerve injury. Glial cells (ie, microglia, astrocytes) increase in number and become activated in the spinal cord in response to intense and/or prolonged pain signals from the periphery after an injury.^35^ This in turn is suspected to play a major role in creating and maintaining peripheral neuropathic pain.

In the CNS, peripheral nerve injury leads to reorganization of neural circuits—characterized by structural and functional changes at multiple levels, including the spinal cord, brainstem, thalamus, and cortex.^36^ The spinal cord undergoes several significant changes aimed at responding to and compensating for the damage. However, some of these changes can contribute to chronic pain and functional deficits, and may persist even after the peripheral injury has healed, leading to long‐term complications. Spinal reflexes are altered after nerve injury with a loss of muscle stretch reflexes and high facilitation of the H reflex and withdrawal reflexes.^37^, ^38^, ^39^ This facilitation of spinal reflexes has been linked to different mechanisms, such as increased synaptic excitability, reduced presynaptic inhibition, and remodeling of sensory afferent projections within the spinal cord.^36^ The normalization of reflex responses correlated with the degree of injury and course of recovery.^38^ Severe injuries further lead to long‐term reorganization of the somatotopy in the spinal cord, leading to a loss of input sensitivity.^40^, ^41^, ^42^ Central sensitization results from sensory neurons in the spinal cord—especially in the dorsal horn—becoming more excitable because of enhanced excitatory glutamatergic signaling and neuropeptide‐induced changes to ion channel expression and axonal sprouting.^43^, ^44^ Apoptosis of inhibitory neurons in the dorsal horn after peripheral nerve injury further contributes to a loss of inhibition, which can lead to an exaggerated response to sensory inputs.^45^ Alterations also take place in cortical and subcortical brain areas. In the somatosensory cortex, the associated cortical map shrank after injury, while receptive fields of other body parts expanded and took over the affected cortical region.^46^, ^47^, ^48^, ^49^, ^50^, ^51^ Transcranial magnetic stimulation (TMS) showed enlarged motor cortical maps and large motor evoked potentials for muscles immediately proximal to the injured nerve in humans.^52^ In case of nerve regeneration, the cortical field may be fully restored.^53^ However, more severe nerve injury involving complete or partial nerve transection is highly likely to result in a permanently distorted cortical map.^48^, ^54^ This pattern of reorganization was also shown for the thalamus and brainstem. Rhizotomies in monkeys led to degeneration of nonnociceptive somatosensory pathways in the brainstem and thalamus, as well as increased activity of thalamic cells innervated by pain afferents.^55^ In animal studies, CNS remodeling began within days after peripheral nerve injury and persisted for months in chronic deafferentation models, with additional remodeling observed up to 7 to 8 months postinjury.^56^, ^57^ In humans, this timeline suggests that remodeling may continue for years after the initial injury.

Spinal cord injuries, like other CNS injuries, elicit a significantly more neurotoxic response compared with peripheral nerve trauma. In the CNS, the immune response to injury is predominantly driven by proinflammatory macrophages; these exert neurotoxic effects as a result of their release of proinflammatory cytokines, proteolytic enzymes, and free radicals.^58^ Wallerian degeneration in the CNS is a markedly more prolonged process compared with the periphery. This delay is partly attributed to the immune response, as well as reduced efficiency of myelinating CNS cells (oligodendrocytes) compared with peripheral nervous system cells (Schwann cells).^59^, ^60^ Overall, a number of factors contribute to a hostile environment for axon regeneration in the CNS. Changes to the supraspinal structures after spinal cord injuries are similar to the ones described for peripheral nerve injuries.^61^, ^62^

**FIG. 1:**
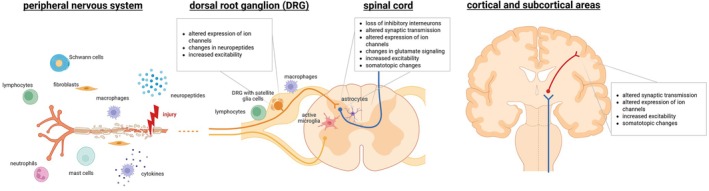
Schematic of the inflammatory processes and plastic changes in the peripheral and central nervous systems after neural trauma. The Wallerian degeneration at the injury site is followed by inflammation and structural remodeling in dorsal root ganglia, spinal cord, and brain. Created with Biorender.com. [Color figure can be viewed at wileyonlinelibrary.com]

**FIG. 2:**
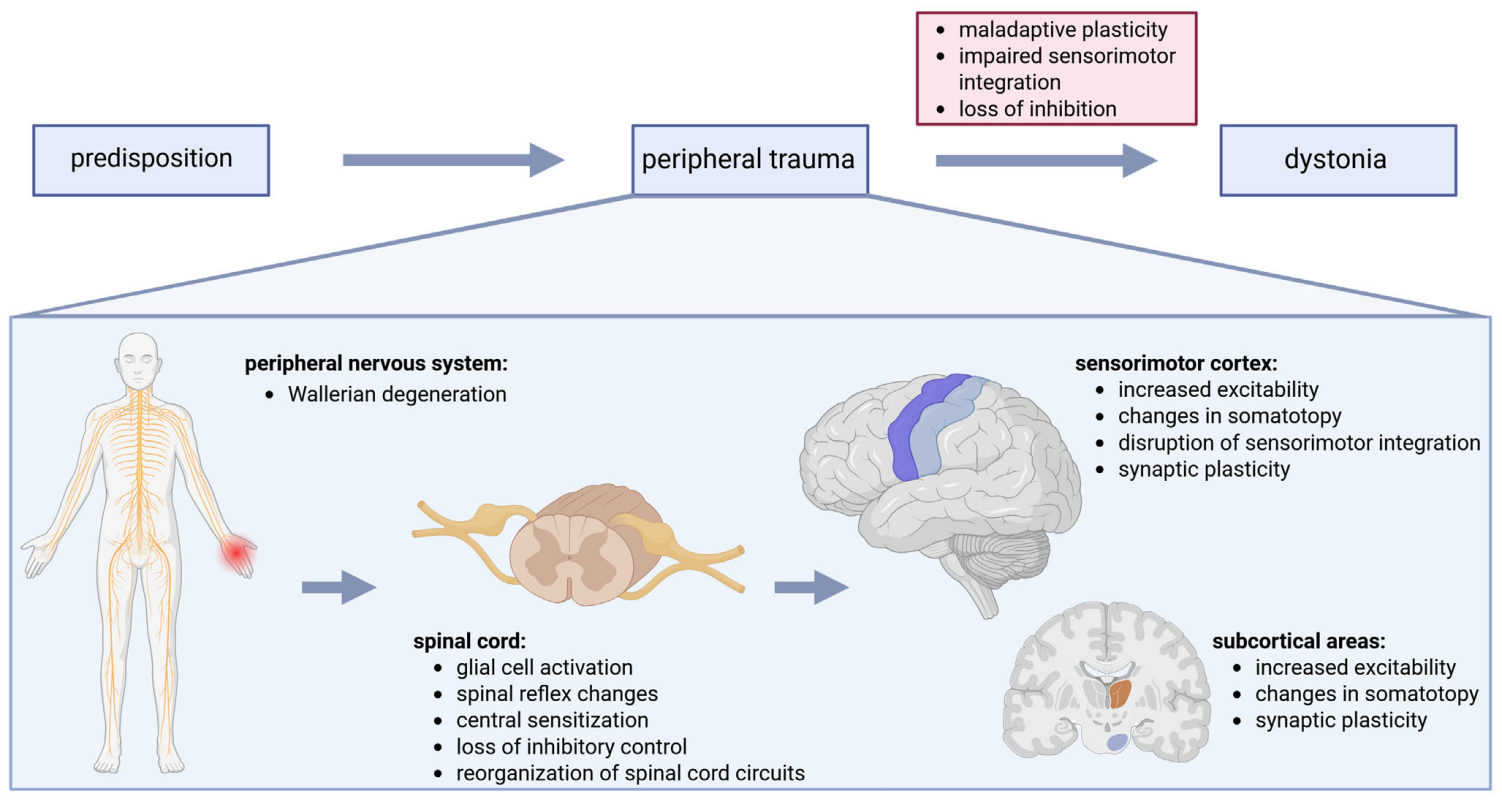
The figure highlights the pathomechanisms behind nerve and spinal cord injury, with reorganization of neural circuits. In the case of genetic predisposition, a peripheral trauma might trigger maladaptive plasticity and lead to the development of dystonia. Created with Biorender.com. [Color figure can be viewed at wileyonlinelibrary.com]

Taken together, sensory deafferentation and motor deefferentation after peripheral nerve injury or spinal cord injury lead to an extensive neuroinflammatory response and remodeling of the CNS (Fig. 1). Although in part necessary for recovery, these responses can become pathological and lead to symptoms such as neuropathic pain, dysesthesia, or even dystonia (Fig. 2).

## Animal Models of Dystonia and the Role of Nerve and Spinal Cord Injuries

### Animal Models Recapitulating Dystonic Phenotypes

Studying animal models that meet both face validity and construct validity is essential when trying to understand the pathophysiological differences between manifesting and nonmanifesting dystonia patients. These differences might contribute to our understanding of how and why dystonic symptoms are triggered in some patients but not others. However, recapitulating the complex phenotype seen in dystonia patients within animal models has consistently proved to be challenging. Rodent models spontaneously developing dystonia‐like symptoms (such as *dt* rat, the *dt* ^*sz*^ hamster, and tottering mice) have been extensively discussed in other reviews.^63^, ^64^ Although they offer robust face validity with emergence of a dystonia‐like phenotype during early development, they fail to fulfill the criteria of construct validity because the genetic basis for the development of the dystonia‐like phenotype is either unknown or mutations in the human homolog are not associated with dystonia.^65^, ^66^, ^67^ An array of drug‐ and lesion‐induced rodent models exhibiting dystonia‐like symptoms have been developed.^68^, ^69^, ^70^, ^71^, ^72^ Although these models have helped identify dystonia as a network disorder, they do not recapitulate the whole dystonia pathophysiology. A large body of genetic animal models for dystonia is available, but most are asymptomatic or develop unspecific, nondystonic motor abnormalities.^73^, ^74^, ^75^, ^76^, ^77^, ^78^ This is surprising, because rodents can in principle develop a dystonia‐like phenotype (eg, as seen in the *dt* rat).

**FIG. 3:**
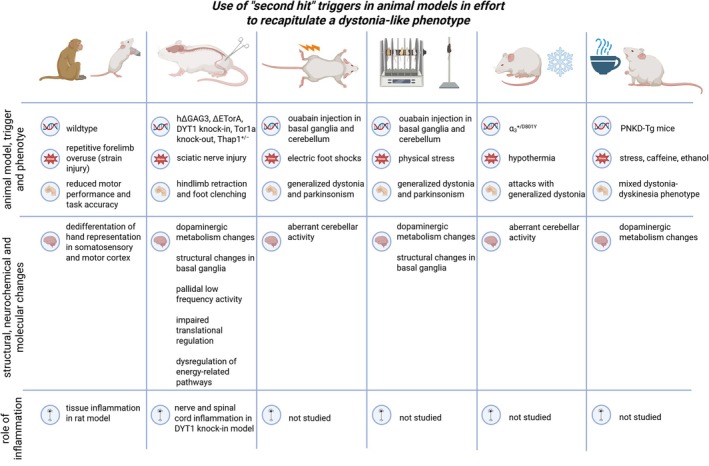
Overview of second hit animal models supporting the role of environmental triggers in dystonia. The figure summarizes changes found in the central and peripheral nervous systems, as well as whether inflammatory processes were analyzed in each model. Created with Biorender.com. [Color figure can be viewed at wileyonlinelibrary.com]

However, there are a few rodent models available that have been shown to recapitulate at least part of the human dystonia phenotype. One genetic mouse model exhibiting dystonia‐like movements is a knock‐in mouse model of levodopa (l‐dopa)‐responsive dystonia, carrying the human p.381Q>K TH mutation.^79^ To model DYT‐TOR1A dystonia, a conditional, biallelic Tor1a knockout gene restricted to the spinal cord and dorsal root ganglia of mice was generated.^9^ All mice developed dystonia‐like symptoms between postnatal days 1 and 3, starting in the hind limbs with a rapid caudorostral spread. A region‐specific deletion of *Tor1a* in GABAergic and cholinergic neurons has also been performed in the forebrain of mice.^80^ Although mice developed dystonia‐like movements, degeneration of cholinergic interneurons was also noted. This has not been observed in DYT‐TOR1A patients, reducing the construct validity of this model. Another approach is to induce a phenotype by applying an extragenetic trigger in genetically predisposed rodents for different forms of dystonia (Fig. 3). These second hit rodent models have been comprehensively reviewed.^26^ For instance, hypothermia induced dystonia‐like attacks in a genetic mouse model for DYT/PARK‐ATP1A3.^81^ In addition, studies in different genetic rodent models for DYT‐TOR1A dystonia showed dystonia‐like movements of the hind limbs after transient denervation by a sciatic nerve crush.^82^, ^83^, ^84^, ^85^, ^86^

### Evidence Supporting the Role of Environmental Triggers in Animal Models

From the second hit perspective, predisposed animal models developing dystonia‐like movements after exposure to an environmental factor have provided important clues about the role of the gene‐environment interaction in dystonia development. Hypothermia, electric stress, physical stress, and peripheral nerve trauma have been described as dystonia triggers in models predisposed for DYT‐TOR1A, DYT‐THAP1, or DYT/PARK‐ATP1A3 dystonia.^81^, ^82^, ^83^, ^84^, ^85^, ^86^, ^87^, ^88^, ^89^ Stress, caffeine, and ethanol were triggers for dyskinesia and dystonia in a mouse model of paroxysmal nonkinesigenic dyskinesia.^90^ Changes to the central motor network correlating with the development of dystonia were shown in all of these models. In our review, the animal models developing dystonia after peripheral nerve injury are of particular importance. The right sciatic nerve was crushed for 1 minute, and animal behavior was observed for up to 12 weeks.^82^, ^83^, ^84^, ^85^, ^86^ Equal nerve regeneration in both DYT‐TOR1A animals and wild‐type animals was ensured via electroneurography. The different symptomatic DYT‐TOR1A rodent models (*Tor1a* knockout mice, hΔGAG3 mice, ΔETorA rats) all demonstrated striatal dopaminergic dysregulation after nerve crush compared with their naive, asymptomatic counterparts and wild‐type animals.^82^, ^83^, ^86^ In the ΔETorA rat model, the dystonia‐like movements were associated with pallidal, low‐frequency activity, which was also reported as a biomarker in patients with dystonia.^82^ Similar to patients with dystonia, dystonic symptoms and pallidal, low‐frequency activity were alleviated by deep brain stimulation of the globus pallidus internus.

In an effort to imitate the suspected trigger(s) behind task‐specific, focal hand dystonias, multiple studies have employed a repetitive, forelimb‐overuse task in either wild‐type monkeys or rats.^91^, ^92^, ^93^, ^94^, ^95^ The validity of these models remains a matter of debate. In one study, some non‐predisposed monkeys demonstrated a reduction in task accuracy and performance speed over time.^91^, ^92^ In a repetitive forelimb task in wild‐type rats, animals displayed reduced motor performance at the end of the overuse period compared with baseline.^94^, ^95^ Clear evidence of dystonia‐like abnormalities in these models was not reported—it is likely that these models represent a model for repetitive strain injury rather than focal hand dystonia, with the reduced performance instead associated with tissue injury or fatigue. Indeed, some studies investigated peripheral changes associated with overuse and found considerable tissue inflammation in the affected forelimb.^94^, ^95^ Similar to models of peripheral nerve injuries, both the affected monkeys and rats developed deterioration and dedifferentiation of the cortical hand representation in the somatosensory and motor cortex after the repetitive forelimb task.^91^, ^92^, ^93^, ^94^

## Human Dystonia and the Relevance of the Second Hit

### Clinical Observations and Epidemiological Studies

In the 1980s and 1990s, many clinical reports described a link between various peripheral traumas and dystonia development (eg, Jankovic and van der Linden,^96^ Schott,^97^ and Sheehy and Marsden^98^). The majority focused on trauma‐induced cervical dystonia.^98^, ^99^, ^100^, ^101^ The reported nature of the trauma was highly diverse, ranging from minor incidents such as slipping on ice resulting in local bruising, to severe injuries such as a pelvic fracture. Similarly, the latency between peripheral trauma and dystonia onset varied widely, spanning from a few hours to as long as 12 months. Some studies have been criticized for potential selection bias.^102^ Although these reports had weaknesses, and from a modern perspective seemed to include patients with functional dystonia, they are certainly intriguing.

In subsequent years, contradictory results concerning the development of dystonia after peripheral trauma were reported. A first case‐control study in Italy saw a positive association between focal body injury and dystonia; another performed on the Italian Dystonia Registry could not reproduce these results.^103^, ^104^ The latter study analyzed a cohort of 1382 patients with idiopathic, adult‐onset dystonia, from which 13 patients reported an injury in the same body part where dystonia later developed—and only in five patients did the injury occur in the year before dystonia development. However, analysis of the medical insurance database in Taiwan found peripheral trauma to be a risk factor for the development of dystonia.^105^ In this study, patients with peripheral trauma had a dystonia incidence of 0.28%, compared with 0.08% in controls; patients with spinal injuries were specifically excluded. Another study suggested that acute peripheral trauma in combination with a sudden onset of dystonia and evidence of fixed dystonia were sensitive and specific predictors for functional dystonia.^106^

Although large‐scale clinical studies are missing for DYT‐TOR1A dystonia, there are a few case reports on dystonia emerging after a peripheral nerve injury or spinal cord injury. DYT‐TOR1A mutation carriers who developed dystonia shortly after a moray bite to the leg, after twisting an ankle, or after surgical removal of an arachnoid cyst at the level of the cervical and thoracic spinal cord have been reported.^5^, ^107^, ^108^ One retrospective review on 28 families found an association between perinatal complications and dystonia development in DYT‐TOR1A mutation carriers.^109^ They could not, however, find an association between physical trauma and dystonia development.

A potential role of peripheral trauma in the development of task‐specific dystonias has not been systematically studied. It is worth debating whether repetitive, prolonged, and stereotyped movement of a limb could be considered a form of mechanical stress or peripheral nerve strain. Although task‐specific dystonias are often broadly characterized as painless, an epidemiological study found that 38% of patients with focal hand dystonia experienced pain.^110^ A relationship between an increase in task performance in the months before the development of task‐specific dystonia has also been described.^111^ Both the report of pain in a subgroup of patients and the increase in task performance reinforce the idea that a focal overuse precedes the symptoms in patients with task‐specific dystonia. However, it must be acknowledged that neither the presence of pain nor the increased task intensity imply the presence of a peripheral nerve injury. Whether this overuse is associated with neuroinflammation has also not been studied in human dystonia patients. Single studies have reported nerve damage in a subgroup of patients with diagnosed task‐specific dystonia. In a case series of 73 patients with musician's dystonia, 40% had ulnar neuropathy.^112^ It has been suggested that a subgroup of patients with writer's cramp may suffer from chronic compartment syndrome.^113^ Overall, these observations remain insufficient to form conclusions on the role of peripheral trauma in task‐specific dystonias.

Epidemiological studies on dystonia development and peripheral or central trauma are extremely sparse; further studies are needed to elucidate the role of peripheral nerve injury and spinal cord injury in dystonia development.

Evidence supporting the involvement of the spinal cord in dystonia development may be derived from acquired forms of dystonia and those linked to nervous system disorders. Indeed, lesions in the cervical spinal cord have been associated with cervical dystonia.^114^ A number of case reports have further highlighted the development of focal forms of dystonia caused by cervical spinal tumors.^115^, ^116^, ^117^, ^118^ Demyelinating disorders affecting the spinal cord, such as multiple sclerosis and neuromyelitis optica spectrum disorders, can further induce dystonic symptoms.^119^ Aside from these reports on acquired forms of dystonia, spinal cord injuries as a potential trigger for dystonia have not been specifically studied.

## Association of Nerve and Spinal Cord Injury with Dystonia

### The Inflammatory Response in Dystonia

A healthy immune system plays a major role in ensuring regeneration after injury, especially in the periphery. Whether the inflammatory response in patients with dystonia is altered compared with healthy controls and whether neuroinflammation itself plays a role in dystonia development have barely been studied. Neuroinflammation has been reported in some of the rarely performed autopsy studies in dystonia. Microgliosis and astrogliosis were shown in postmortem brains of patients with X‐linked dystonia‐parkinsonism.^120^ It is important to note, however, that X‐linked dystonia‐parkinsonism is a neurodegenerative disorder—a characteristic not typically associated with most other forms of dystonia. Increased and enlarged astrocytes were found in the basal ganglia in an autopsy study of a 35‐year‐old DYT/PARK‐ATP1A3 patient.^121^ For spasmodic dysphonia, small clusters of activated microglia and macrophages were found in the brainstem of two patients compared with four healthy controls.^122^ No signs of inflammation were found in an autopsy study of DYT‐TOR1A patients.^123^ Interestingly, markers for inflammation were identified in a subgroup of patients with cervical dystonia,^124^ where 40% of included cervical dystonia cases presented with abnormal immune cell frequencies. More than one‐third of the cervical dystonia cases had increased B cells and monocytes, the distribution of CD8^+^ cells to CD4^+^ cells was bimodal compared with healthy controls, and one‐third of cases had relatively increased CD8^+^ T cells and relatively decreased CD4^+^ T lymphocytes. Another study found a decrease in T lymphocytes in 11 patients with cervical dystonia compared with healthy controls.^125^ A proteomics study on 100 individuals with cervical dystonia observed abnormalities in pathways correlated to immune‐related processes.^126^ Another recent proteomics study of patients with cervical dystonia, laryngeal dystonia, and blepharospasm also determined that the top biological pathways were related to immune system changes.^127^ The top three enriched Gene Ontology terms were pathways associated with negative regulation of the immune system processes, leukocyte migration, and interleukin‐10 production. Cervical dystonia has also been reported in association with autoimmune disorders.^128^, ^129^, ^130^ These studies on cervical dystonia and other forms of adult‐onset focal dystonia are intriguing and suggest potential dysfunction of the immune system in at least a subgroup of patients with dystonia. However, further clarification on the pathomechanisms behind this neuroinflammation and the causative link to dystonia is necessary.

To our knowledge, no studies in patients with dystonia have investigated the neuroinflammatory response after peripheral nerve injury or spinal trauma. In *Tor1a* knockout mice subjected to a peripheral nerve crush, no differences were found for CD11b^+^ microglial cells in the spinal cord 8 weeks post–nerve crush.^86^ However, in a DYT1 knock‐in model, CD11b^+^ cells were significantly increased in both the gray and white matter of the spinal cord and in the crushed sciatic nerve 12 weeks post–nerve trauma in the genetically mutated animals, but not in their naive counterpart.^131^ CD4^+^ T cells were also increased in the affected sciatic nerve of genetically mutated animals compared with controls, while CD8^+^ T cells were increased in both DYT‐TOR1A and wild‐type animals after nerve trauma. No differences were found concerning the immune cells in the striatum after the nerve crush. Although these findings could indicate an altered and prolonged inflammatory response to peripheral nerve injury, replication and further investigation into the mechanisms behind it are required.

Complex regional pain syndrome (CRPS) can in some cases be associated with fixed dystonic postures. CRPS can result from peripheral trauma without (CRPS I) or with (CRPS II) major peripheral nerve damage, with dystonia seeming to be more common in CRPS I.^132^ To date, it remains unclear why some patients develop dystonia, whereas others do not. Pathomechanisms in CRPS are believed to involve a disturbed immune response with a proinflammatory state, both in the periphery and the CNS.^133^ A significant role in CRPS development is assigned to neurogenic inflammation entailing the activation of nociceptive receptors—in turn, this facilitates the release of neuropeptides and leads to a number of inflammatory reactions such as the release of proinflammatory cytokines.^134^, ^135^ A disrupted equilibrium between proinflammatory and anti‐inflammatory cytokines, along with the involvement of microglia and astrocytes in the CNS, are additional suspected pathomechanisms.^136^, ^137^ Typical focal dystonia and fixed dystonic postures after CRPS are probably distinct disease entities, and their pathogenesis very likely differs. However, it is possible that shared inflammatory mechanisms could contribute to dystonic symptoms in cases where dystonia is associated with peripheral nerve injury—but again, further research is needed.

Severe injuries can also encompass the development of depression, anxiety disorders, or posttraumatic stress disorder (PTSD), such as in military‐related injuries.^138^, ^139^ Neuroinflammatory mechanisms play a major role in these disorders, with both PTSD and depression associated with elevated proinflammatory cytokines and microglial dysfunction.^140^, ^141^ These psychological stressors could have an additional impact on the development of dystonia after neural injury. Psychological stress has been reported to be a trigger for dystonia in a small number of studies and case reports.^142^, ^143^, ^144^ However, a potential link between neural injury, the development of PTSD or depression, and dystonia remains to be studied.

### Remodeling of the CNS in the Dystonic Brain After Peripheral Nerve or Spinal Cord Injury

Similar to patients with peripheral nerve injury, disorganization of the somatosensory presentation has been observed in patients with focal dystonia.^145^, ^146^ Enhanced sensorimotor plasticity is one of the widely accepted pathomechanisms behind dystonia.^147^ Multiple studies have evidenced increased plasticity via noninvasive neuromodulation techniques and neuroimaging.^148^, ^149^, ^150^ Experiments involving noninvasive neuromodulation techniques also indicated impaired homeostatic plasticity in patients with dystonia.^151^ Indeed, a protocol combining low‐frequency, repetitive TMS (rTMS) and transcranial direct current stimulation (TDCS) to study homeostatic plasticity of the motor cortex showed drastically different response patterns in patients with writer's cramp compared with controls.^151^ After preconditioning with TDCS, subsequent low‐frequency rTMS did not enhance the inhibitory effect on corticospinal excitability in patients with dystonia, contrary to the findings in healthy controls. The authors concluded that the homeostatic mechanisms limiting excitability levels were impaired in patients with dystonia. Considering these findings, it is conceivable that the combination of an external trigger (such as a trauma known to lead to remodeling of the CNS) with pathologically altered neural plasticity that is easy to induce, as well as excessive, leads to dystonia development in a predisposed brain.

Aside from neuroinflammation, a peripheral nerve injury or spinal cord injury is accompanied by pain. This raises the question whether pathological processing of sensory information, particularly pain, might also contribute to dystonia development.^152^, ^153^ The role of pain itself as a potential second hit is understudied in dystonia. Indeed, disease‐related pain has been described in a remarkable percentage of patients with dystonia—up to 90% in patients with cervical dystonia and 38% in patients with focal hand dystonia.^110^, ^154^, ^155^ However, sensory symptoms have also been described, such as pain in the neck muscles before the development of cervical dystonia, dry eye syndrome before the development of blepharospasm, and irritation of the throat before the development of spasmodic dysphonia.^98^, ^156^ In a child with a DYT‐TOR1A mutation, dystonia developed after a moray bite—trauma described as mild, but which caused severe pain in the patient.^108^ Studies have described alterations in nociceptive processing of painful stimuli in patients with dystonia.^157^, ^158^ Others have hypothesized that alterations of the somatosensory cortex in patients could represent a potential risk factor for the development of dystonic pain because of abnormal processing of sensory information.^152^, ^159^, ^160^ Overall, these findings highlight the need for further studies, because it remains unresolved whether pain resulting from a trauma could be part of a trigger mechanism.

## Challenges and Future Directions

A major challenge in unraveling dystonia pathogenesis continues to be the collection of reliable data. To enable a clear, causative link between peripheral nerve injuries or spinal cord injuries and dystonia to be established, large, prospective clinical studies are necessary. Current studies suffer from an array of weaknesses, such as retrospective assessments liable to recall bias, small study populations, and a highly variable consideration of time spans between trauma and dystonia onset.^26^ For future studies, a detailed assessment of the trauma—including data on the extent of nerve or spinal cord damage, the presence of neuroinflammation, and the follow‐up treatment—will be indispensable. It remains to be resolved whether peripheral or spinal trauma before dystonia development even needs to involve electrophysiologically measurable neuronal damage. A clinical evaluation of dystonic symptoms—with consideration of whether it is phasic or tonic dystonia, whether it is focal or spreads from the initial site of onset, and whether there is a genetic predisposition—will also be necessary. These assessments are especially important because tonic dystonia after a peripheral nerve trauma has been discussed as potentially having a functional background.^106^ Indeed, available studies make it difficult to differentiate between functional dystonia, acquired dystonia, and inherited dystonia triggered by an extracranial trauma.

If a causative link can be established for trauma and dystonia for a subgroup of patients, this would highlight the importance of considering a second hit in dystonia development *and* help to develop therapeutic strategies. These therapeutic options could include early treatment of neuroinflammation and pain to potentially attenuate dystonia development. Noninvasive brain stimulation in patients with focal hand dystonia has also been studied as a possibility to treat the pathological, cortical alterations in patients with dystonia.^161^ To name a few examples, rTMS protocols have been applied to reverse the abnormal cortical excitability found in patients with dystonia, with promising results.^149^, ^162^ Others have used proprioceptive training in the form of vibration in an effort to restore sensorimotor organization in patients with focal hand dystonia.^163^

## Conclusion

Existing second hit animal models provide evidence supporting the development of dystonia after peripheral nerve trauma in individuals with a genetic predisposition for DYT‐TOR1A dystonia,^82^, ^83^, ^84^, ^85^, ^86^ as shown by genetically mutated DYT‐TOR1A rats and mice developing dystonia‐like movements of the hind limb during the recovery period after a sciatic nerve crush injury. Pathological findings in the central motor network of these models have shown clear genotype‐ and phenotype‐dependent differences. However, it remains challenging to identify the causative mechanisms behind the development of dystonia‐like movements after trauma. At this stage, it is unclear whether neuroinflammatory processes contribute to this development. Further, it remains to be studied whether dystonia is triggered by an abnormal sensory input or an abnormal brain response to the sensory input of the nerve injury. Although spinal cord injuries per se have not been analyzed in animal models for dystonia, a recent study using a conditional, biallelic *Tor1a* knockout gene restricted to the spinal cord and dorsal root ganglia of mice identified the spinal cord as a potential major player in dystonia development in DYT‐TOR1A dystonia.^9^ Overall, animal models have shown a link between peripheral trauma and dystonia development—however, this association is less clear in studies with human dystonia patients. Nonetheless, strong indicators exist for the role of environmental factors in human dystonia, including reduced penetrance in monogenic dystonias and the triggers reported for certain forms of dystonia, such as DYT/PARK‐ATP1A3. This warrants a closer look at the role of nerve and spinal cord injury as potential triggers in a subgroup of patients with dystonia, because this will encourage new therapeutic strategies for a disease where treatment options are still very limited.

## Author Roles

(1) Research Project: A. Conception, B. Organization, C. Execution; (2) Statistical Analysis: A. Design, B. Execution, C. Review and Critique; (3) Manuscript Preparation: A. Writing of the First Draft, B. Review and Critique.

L.H.‐R.: 1B, 3A.

C.W.I.: 1A, 1B, 3B.

## Financial Disclosures of All Authors (for the Preceding 12 Months)

L.H.‐R. and C.W.I. are employed by the University Hospital Würzburg. C.W.I. receives funding from the Interdisciplinary Center for Clinical Research (IZKF) at the University of Würzburg (A‐303, A‐421, and N‐362). L.H.‐R. and C.W.I. receive funding from the Deutsche Forschungsgemeinschaft (DFG, German Research Foundation) Project‐ID 424778381‐TRR 295 (A01, A06, and A07). C.W.I. is funded by the VERUM foundation. C.W.I. reports consultancies and honoraria from Teva, Merz, Ipsen, and AbbVie. He is listed as an inventor on patent WO2023180548A1.

## Data Availability

Data sharing not applicable to this article as no datasets were generated or analysed during the current study.
